# Shift work and pathological conditions

**DOI:** 10.1186/1745-6673-1-25

**Published:** 2006-12-11

**Authors:** Anke van Mark, Michael Spallek, Richard Kessel, Elke Brinkmann

**Affiliations:** 1 Institute of Occupational Medicine, University of Lübeck, D-23538 Lübeck, Germany; 2 Department of Occupational Medicine, VW-Nutzfahrzeuge, D-30405 Hannover, Germany; 3 Department of Prevention, Norddeutsche Metall-Berufsgenossenschaft, D-30173 Hannover, Germany

## Abstract

Shift work exerts major influences on the physiological functions of the human body. These are primarily mediated by the disruption of circadian rhythms since most body functions are circadian rhythmic. Next to the disturbances caused by changes in the circadian system, shift work has also been suggested to be related to a number of other health disorders. The present study summarizes recently published data on the potential relationship between disorders and shift working.

## Background

Occupational and environmental medicine cover a broad field of areas including respiratory disorders [1-6], haematological disorders [7], musculoskeletal disorders [8,9] and dermatological and rheumatic disorders [10,11]. Shift work related disorders are common medical problems and are encountered daily throughout the world by many practitioners. Therefore, a distinct field of research has focused on the identification of specific shift work-related issues [12-15]. One of the most important areas of shift-work research is the characterization of pathways by which shift work can exerts its influence on human health [16-19]. In this respect, nearly all biological functions beginning on the subcellular level have a circadian rhythm [20,21]. It has become obvious that even disturbances of single aspects of these rhythms may lead to major effects [22-24]. The present review summaries recently published data on the association of shift work with different diseases.

## Methods

A PubMed research was performed using the terms "shift", "work", and "shift work" and publication types (date: 2006-01-04). Articles were screened for their contents and relevant data was analysed.

### Number of publications related to shift work

**Figure 1:**
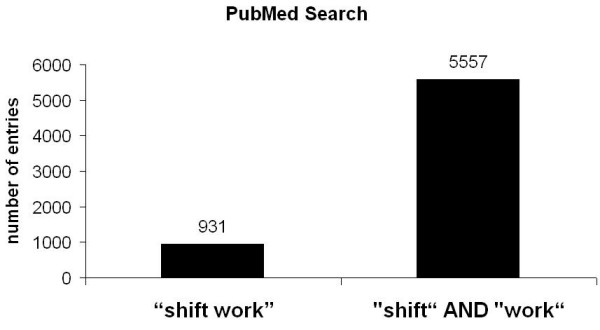
PubMed search for the terms "shift work" and "shift" and "work".

**Figure 2:**
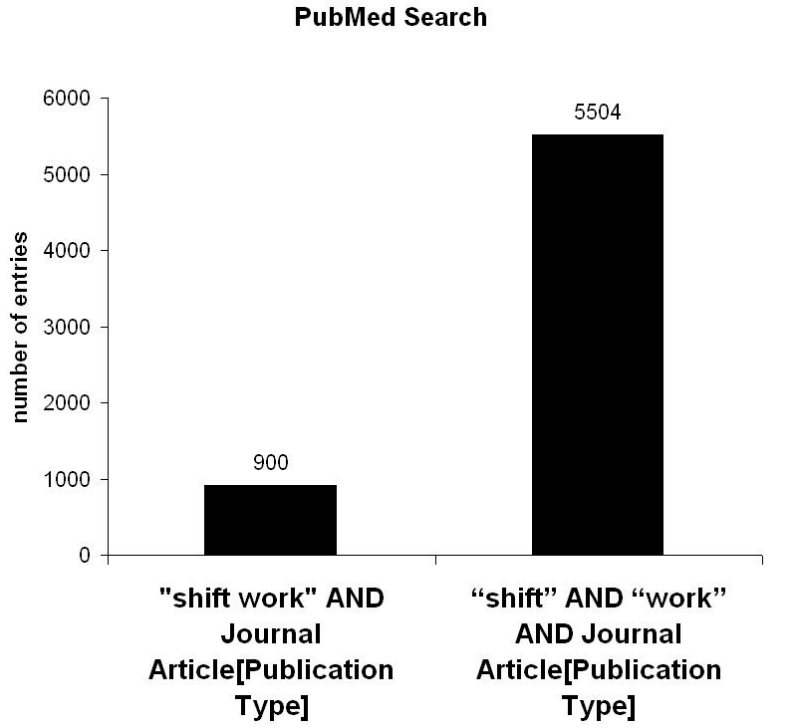
PubMed search for the terms "shift work" and "shift" and "work" and publication type "journal article".

**Figure 3:**
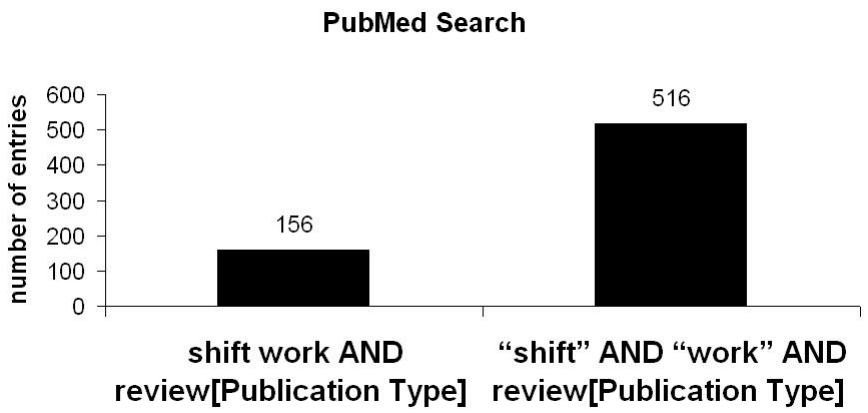
PubMed search for the terms "shift work" and "shift" and "work" and publication type "review".

For the term „shift" 93718 entries were registrated. To analyse specific articles related to shift work, the search was narrowed and for the terms „shift" AND „work" 5557 entries were found. To further delineate the research the term "Shift work" was entered and 931 entries were registered which were analysed for their contents (fig. 1). For the terms "Shift" AND "work" AND "Journal Article" [Publication Type] were 5504 and for the terms "Shift work" AND "Journal Article" [Publication Type] 900 entries were listed (fig. 2). Also, reviews related to the matter were analysed and 516 entries were found for "Shift" AND "work" AND "review" [Publication Type] and 156 entries for "Shift work" AND "review" [Publication Type] 156 (fig. 3).

### Potential association between shift work and having a metabolic syndrome

Since shift work has been related to an increased risk of cardiovascular diseases metabolic risk factors like obesity, elevated lipid levels, or disturbed glucose tolerance and not least the sum the metabolic syndrom between shift workers were focused in recent studies [16,25]. Since causal pathways for this association are only partly known, Karlsson et al. studied a working population of 27,485 people from the Vasterbotten intervention program (VIP) [26]. From this population cross sectional data, including blood sampling and questionnaires were collected via a health survey. The authors reported that obesity was more prevalent among shift workers in all age strata of women. By contrast it was more prevalent only in two out of four age groups in men [26]. It was also found that an increase in triglyceride levels (>1.7 mmol/l) was more common among two age groups of shift working women while no significant differences were present among men. In the youngest male and female age groups of shift workers low concentrations of high density lipoprotein cholesterol were recorded while an impaired glucose tolerance was found more often among 60 year old female shift workers [26]. After the adjustment for age and socioeconomic factors, both obesity and high triglycerides persisted as risk factors in male and female shift workers (OR of 1.4 for obesity and 1.1 for high triglyceride concentrations) [26]. It can be summarized that in this population cross sectional study, obesity, low concentrations of high density lipoprotein cholesterol, and high triglycerides, seem to be present more frequently in shift workers. This may suggest an association between metabolic syndrome and shift work.

### Shift work and coronary heart disease

An association between shift work and myocardial infarction has been postulated since many years [27,28]. In this respect, the risk to develop coronary heart disease may be due to job strain and there might be an interaction between shift work and job strain influencing the development of coronary heart disease. Knutsson and colleagues performed a study in order to assess the relation between shift work, job strain, and coronary heart disease. They compared 2006 cases with acute myocardial infarction to 2642 controls without symptoms of myocardial infarction in a population based case-control study [29]. It was shown that myocardial infarction risk was associated with shift work both in men and women (odds ratio (OR) 1.3, 95% confidence interval (95% CI) 1.1 to 1.6 and OR 1.3, 95% CI 0.9 to 1.8, respectively). Pronounced values were found for the age group 45–55 with a relative risk being 1.6 for men and 3.0 for women. No interactions were found between shift work and job strain. It may be concluded that shift work is associated with myocardial infarction in both men and women. No relation was found with job strain, smoking, or job education level. This may indicate that the precise mechanisms are still not clear[29].

### Oxidative stress and shift work

Antioxidant capacity has been related to numerous diseases and antioxidant enzymes exert major effect on various cellular functions [30-32]. It may also be influenced by shift work. To analyse the effects of night-shift working on the antioxidant capacity Sharifian et al. performed a study in 44 workers with a rotational shift schedule [33]. They had a mean age of 36.57 years (SD: 10.18) and mean BMI of 26.06 (SD: 4.37). Two blood samples were taken from the participants, one after their day shift and one after their night shift [33]. The total plasma antioxidant capacity of each subject was analysed and it was shown that the total plasma antioxidant capacity was measured in 44 shift-workers after their day and night shifts. The mean reduction of total plasma antioxidant capacity after the night shift was 105.8 μmol/L (SD: 146.39) [33]. A significant correlation was found between age and weight and total plasma antioxidant capacity. Also, age and weight were reported to be inversely related to the total plasma antioxidant capacity. The authors concluded that shift work may exert negative influences on the total antioxidant capacity and may therefore be regarded as an oxidative stressor. Also, factors such as aging and obesity makes shift workers more sensitive to this hazardous effect which may be a key mechanism for the detrimental effects of shift working [33].

### Common infections and shift work

As suggested by a lack in the antioxidative capacity, shift work may also have influence on the prevalence of infections [34]. To analyse these effects Mohren et al. studied the prevalence of common infections among employees in different work schedules [35]. For collection of data the authors used self-administered questionnaire data from the Maastricht Cohort Study on "Fatigue at Work" (n = 12.140). As matching variable between day and shift workers to control for their different work environment job title was used. A multilevel analysis of a two-level structure was performed, in which the individual employees (level 1) were nested within job titles (level 2), adjusted for demographics, longstanding disease, health behavior, work-related factors, fatigue and sleep quality [35]. It was revealed that shift work was associated with a higher risk for common infections compared to day work. The highest risk was present in three-shift workers. In comparison to day work, shift work was also associated with differences in health, health behavior, sleep, fatigue and perceived job characteristics. The authors proposed that these factors may also influence the occurrence of infections [35].

### Night shifts and breast cancer risk

Megdal et al. examined the association between night shift work and breast cancer risk by performing a meta-analysis of observational studies to assess the effects of night work on breast cancer risk. They screened the PubMed from January 1960 to January 2005 using search terms such as night work terms, flight personnel terms, cancer terms, and risk terms and performed independent data extraction by two authors using standardised forms [36]. Based on 13 studies, including seven studies of airline cabin crew and six studies of other night shift workers the authors reported an aggregate estimate for all studies combined of 1.48 (95% CI, 1.36–1.61). They found a similar significant elevation of breast cancer risk among female airline cabin crew (standardised incidence ratio (SIR), 1.44; 95% CI, 1.26–1.65), and female night workers (relative risk (RR), 1.51; 95% CI, 1.36–1.68) separately [36]. They also found evidence suggesting confounding due to incomplete adjustment for breast cancer risk factors, with smaller effects in the studies that more completely adjusted for reproductive history and other confounding factors. No significant asymmetry (P > 0.05) was present in the Egger's and Begg and Mazumdar's tests for publication bias. It may be concluded that the presently published studies on night shift work and breast cancer risk collectively point to an increased breast cancer risk among women [36].

Several studies were performed in Seattle to investigate the effects of factors that can disrupt circadian rhythm and alter normal nocturnal production of melatonin and reproductive hormones of relevance to breast cancer etiology [37]. Studies completed demonstrated that: 1. an increased risk of breast cancer associated with indicators of exposure to light-at-night and night shift work [37]; 2. decreased nocturnal urinary levels of 6-sulphatoxymelatonin associated with exposure to 60-Hz magnetic fields in the bedroom the same night, and a number of other factors including hours of daylight, season, alcohol consumption and body mass index [37].

## Conclusion

**Figure 4:**
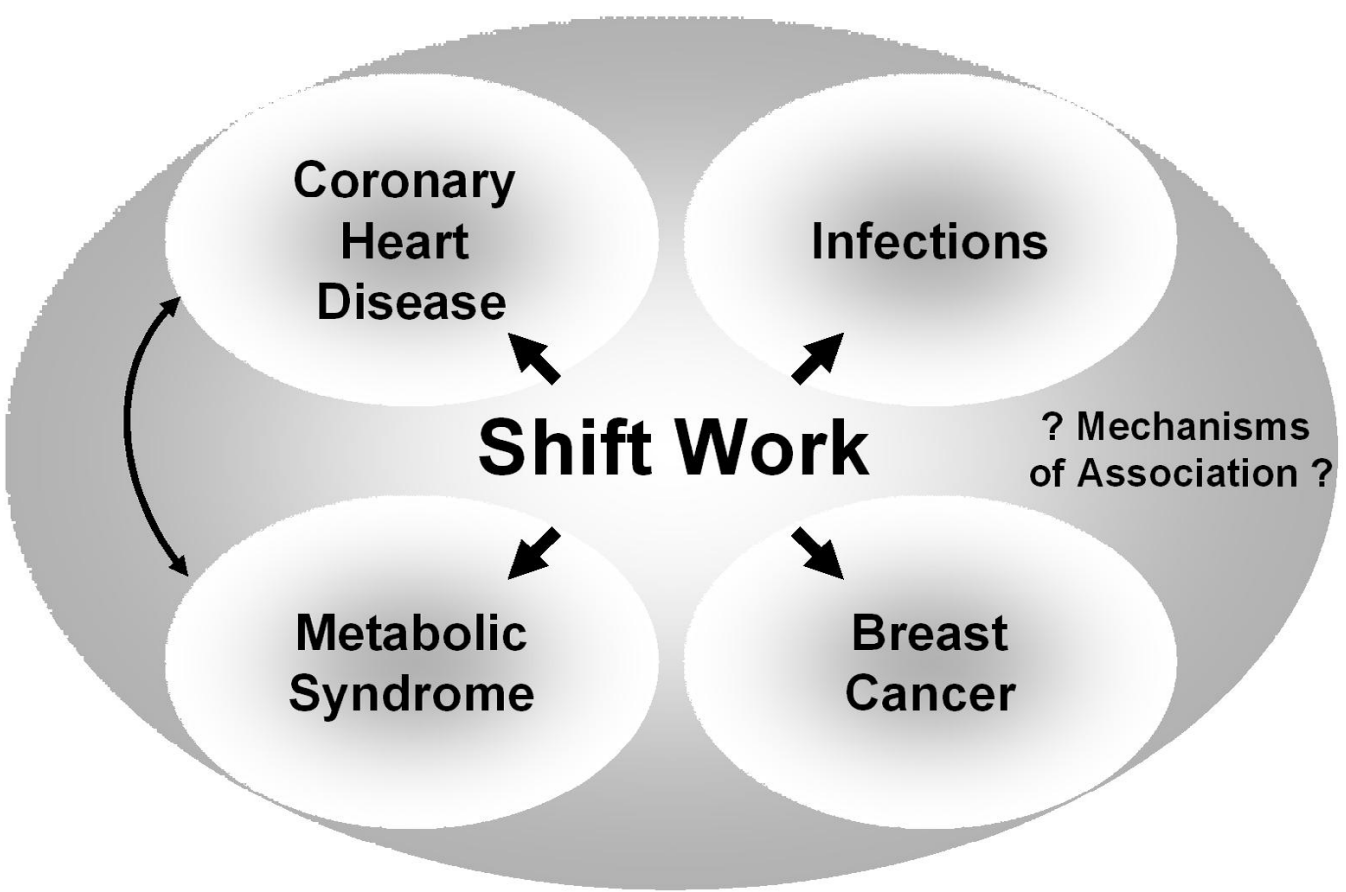
Potential associations of shift work and medical disorders.

There is a large amount of data pointing to an association between shift work and the prevalence of many medical conditions. However, as these disorders are often based on a variety of non-occupational factors, a distinct separation into either occupational (shift-work-related) or non-occupational can be difficult (fig. 4). For example metabolic disturbances are caused by the disruption of circadian rhythms or by lifestyle of shift workers? Future experimental and epidemiological studies have to bring a better understanding of the factors influenced by shift work. Therefore, next to enlarging the epidemiological knowledge, experimental studies encompassing modern techniques from molecular biology [38-41], physiology [42-46], morphology [47-49] and toxicology [50] should be used to identify further cellular mechanisms.

## Declaration of competing interests

The author(s) declare that they have no competing interests.

## Authors' contributions

AvM, LBC, RK, and EB have all been involved in drafting the article or revising it critically for important intellectual content and have given final approval of the version to be published.
